# Infectious diseases, cardio-cerebrovascular health and vaccines: pathways to prevention

**DOI:** 10.1007/s40520-025-02968-y

**Published:** 2025-03-13

**Authors:** Fiona Ecarnot, Jotheeswaran Amuthavalli Thiyagarajan, Mario Barbagallo, Jane Barratt, Tor Biering-Sørensen, Elisabeth Botelho-Nevers, Marco Del Riccio, Marco Goeijenbier, Stefan Gravenstein, Luis Lourenço, Jean-Pierre Michel, Daniela Pedicino, Cornel Sieber, Antoni Torres, Nicola Veronese, Massimo Volpe, Thomas Weinke, Stefania Maggi

**Affiliations:** 1 SINERGIES Unit, University Marie & Louis Pasteur, 19 rue Ambroise Paré, Besançon, 25000 France; 2 Department of Cardiology, University Hospital Besancon, 3-8 Boulevard Fleming, Besancon, 25000 France; 3 Responsible officer for Bone Health and Ageing Initiative, Ageing and Health Unit, Department of Maternal, Adolescent Health and Ageing, World Health Organization, Newborn, Child, Geneva, Switzerland; 4 Geriatric Unit, Department of Internal Medicine and Geriatrics, University of Palermo, Via del Vespro 141, Palermo, 90127 Italy; 5 International Federation on Ageing, Toronto, Canada; 6 Center for Translational Cardiology and Pragmatic Randomized Trials, Department of Biomedical Sciences, Faculty of Health and Medical Sciences, University of Copenhagen, Copenhagen, Denmark; 7 Cardiovascular Non-Invasive Imaging Research Laboratory, Department of Cardiology, Copenhagen University Hospital - Herlev and Gentofte, Copenhagen, Denmark; 8 Steno Diabetes Center, Copenhagen, Denmark; 9 Department of Cardiology, Copenhagen University Hospital - Rigshospitalet, Copenhagen, Denmark; 10 Infectious Diseases Department, University Hospital of Saint-Etienne, Saint-Etienne, 42055 cedex France; 11 Team GIMAP, CIRI– Centre International de Recherche en Infectiologie, Université Jean Monnet, Université de Lyon, Inserm, U1111, CNRS, UMR530, Saint-Etienne, 42023 France; 12 Department of Health Sciences, University of Florence, Florence, 50134 Italy; 13 Department of Intensive Care, Spaarne Gasthuis, Haarlem, The Netherlands; 14 Division of Geriatrics and Palliative Medicine, Department of Medicine, Alpert Medical School of Brown University, Providence, RI USA; 15 International Pharmaceutical Federation (FIP), The Hague, The Netherlands; 16 University of Geneva, Geneva, Switzerland; 17 Fondazione Policlinico Universitario “A.Gemelli”- IRCCS, Catholic University of the Sacred Heart, Rome, Italy; 18 Institute for Biomedicine of Aging, Friedrich-Alexander University, Erlangen-Nürnberg, Germany; 19 County Hospital, Ciberes, Switzerland; 20 University of Barcelona, Institut d´Investigacions Biomédiques August Pi i Sunyer (IDIBAPS), Ciberes, Barcelona Spain; 21 Saint Camillus International University of Health Sciences, Rome, Italy; 22 SIPREC, Italian Society for Cardiovascular Prevention, Rome, Italy; 23 IRCCS San Raffaele Roma, Rome, Italy; 24 Clinical Practice, Infectious diseases and Gastroenterology, Berlin, Germany; 25 National Research Council, Neuroscience Institute, Aging Branch, Padova, Italy

**Keywords:** Cardiovascular, Cerebrovascular, Influenza, SARS-CoV-2, COVID, Respiratory syncytial virus, Herpes zoster, Pneumococcal disease, Vaccination

## Abstract

Cardiovascular and infectious diseases both feature among the leading causes of death among men and women in the world. The pathophysiological pathways of infection and cardiovascular disease intersect, and there is a bidirectional relationship between the two. Vaccines are available for the most common infectious diseases affecting older adults, such as influenza, pertussis, pneumococcal disease, herpes zoster, COVID and respiratory syncytial virus (RSV). In many countries, these vaccines are recommended systematically for older adults and any adults with comorbidities, who are also those most likely to suffer from cardiovascular disease. There is a large body of evidence attesting to the benefits of vaccination on cardio- and cerebrovascular health. The European Interdisciplinary Council for Aging (EICA) and the Italian Society for Cardiovascular Prevention (Società Italiana per la Prevenzione Cardiovascolare, SIPREC) convened a 2-day meeting in June 2024 to review the state of the evidence on the relationship between cardio- and cerebrovascular health and the most common infectious diseases, and the role of vaccines in preventing both infection and its adverse consequences in terms of cardiovascular and cerebrovascular outcomes. We present here the Executive Summary of the proceedings of this meeting.

## Introduction

Cardiovascular disease is the leading cause of death among men and women in today’s society, accounting for 13% of all deaths in the world [1]. Infectious diseases also figure prominently among the top ten causes of death, notably COVID-19 and lower respiratory tract infections [2]. The pathophysiological pathways of infection and cardiovascular disease intersect, and there is a bidirectional relationship between the two. Indeed, the mechanisms of acute infection may increase the short term risk of adverse events in patients with cardiovascular disease [3, 4], while conversely, patients with cardiovascular disease are at increased risk of infection [5, 6]. One of the likely mechanisms behind this reciprocal relation is systemic inflammation, a pathogenic state that impacts an individual’s immune function and immune response, thereby impacting the ability to mount a robust defence against infection [7, 8]. Vaccines are available for the most common infectious diseases affecting older adults, such as influenza, pertussis, pneumococcal disease, herpes zoster, COVID and respiratory syncytial virus (RSV). In many countries, these vaccines are recommended systematically for older adults and any adults with comorbidities, who are also those most likely to suffer from cardiovascular disease. There is a large body of evidence attesting to the benefits of vaccination on cardio- and cerebrovascular health.

In this context, the European Interdisciplinary Council for Aging (EICA) and the Italian Society for Cardiovascular Prevention (Società Italiana per la Prevenzione Cardiovascolare, SIPREC) felt that there was a compelling need to review the state of the evidence on the relationship between cardio- and cerebrovascular health and the most common infectious diseases, and the role of vaccines in preventing both infection and its adverse consequences in terms of cardiovascular and cerebrovascular outcomes. To this end, EICA and SIPREC jointly convened a 2-day in person and virtual meeting on 10–11 June 2024. We present here the Executive Summary of the proceedings of this meeting. Speakers were tasked with summarizing the current state of knowledge on each specific topic, in particular identifying knowledge gaps that could be avenues for future collaborative research.

Advances in our understanding of inflammation and atherosclerosis have established unequivocally that atherosclerosis is an inflammatory disease [9, 10]. Acute coronary events characterized by rupture of a coronary plaque rich in “red” thrombus and macrophages, against a background of systemic inflammation with high C-reactive protein (CRP), are the most strongly associated with inflammation [11], which in turn is associated with frailty in older adults [12]. Current evidence suggests that the inflammatory pathway could be targeted in atherosclerosis as a means to reduce the risk of cardiovascular events [13, 14]. Infection is thought to be one potential non-canonical risk factor for atherosclerosis [15]. Indeed, influenza and other respiratory tract infections may promote systemic inflammation and prompt plaque destabilization [16]. Among the common respiratory infections, influenza is the most widely studied due to the strong relation that has been established with cardiovascular disease, especially ischaemic and myocardial complications such as myocardial infarction, stroke, or heart failure [6]. The general effects induced by influenza infection are shared with other viruses and pathogens, and these include the generation of a thrombogenic environment by the release of procoagulant factors, and increased platelet reactivity, leading to a higher risk of thromboembolic events. Several studies have linked influenza infection with an increased risk of cardiovascular events [17–20]. A self-controlled case-series study by Kwong et al. [20] showed that within the first 7 days after a positive influenza test, the risk of AMI was 6 times higher than at any other timepoint. Respiratory tract infection has also been reported to be associated with a 2.5-fold increase in the risk of stroke [18], while influenza-like illness is also temporally associated with an increased risk of hospitalization for heart failure [19].

Amongst other pathogens associated with cardiovascular events, there is *Streptococcus pneumoniae*, the leading cause of community-acquired pneumonia (CAP) [21], with a high burden of disease in older adults in particular. The systemic inflammatory response to respiratory infection, e.g. influenza or pneumonia, can prompt acute cardiovascular events in patients with a history of cardiovascular disease or ongoing atherosclerosis. The augmented risk due to pneumonia infection may persist for several weeks or months, while an increased risk after pneumonia with sepsis may persist for up to several years [4]. An estimated 23% of patients with invasive pneumococcal disease suffer a major adverse cardiovascular event, and 28% of patients hospitalized for CAP [22].

Respiratory Syncytial Virus (RSV) is another virus with high circulation that causes respiratory infections. Indeed, without laboratory testing, it is impossible to distinguish RSV infection from other respiratory viruses clinically. As with the other viruses mentioned, the relation between RSV and CVD is bidirectional, involving mechanisms such as inflammation, proinflammatory cytokines that can disrupt endothelial function, a hypercoagulable state with its risk of thrombosis, and plaque destabilization and rupture in patients with a predisposition. As with all infections, fever can also increase risk of arrhythmia. SARS-CoV-2 infection creates both arterial and venous side effects, including endothelial dysfunction and immune dysregulation, leading to an inflammatory and hypercoagulable state that affects both the macro- and the microvasculature [23]. Clinically, COVID-19 can lead to thromboembolic events, cardiovascular manifestations, impaired arterial stiffness, cerebrovascular complications, nephropathy, or retinopathy, especially after severe illness, and effects may persist for months or years [24].

Respiratory infections are an important trigger for exacerbations of chronic respiratory conditions such as chronic obstructive pulmonary disease (COPD). Pneumococcal and viral respiratory infections, such as influenza, pertussis, RSV and COVID 19, increase exacerbations, hospitalisation and mortality in patients with COPD [25], but vaccination helps to reduce risk of hospitalization, cardiovascular events, and exacerbations in COPD patients [26–28].

There is also a bidirectional relationship between respiratory infections, more specifically influenza, and diabetes [29]. Overall, persons living with diabetes are at higher risk of infection, and once infected, the clinical course of infections tends to be more severe, resulting in higher morbidity and mortality rates. On the other hand, influenza, or any other respiratory virus infection, could worsen diabetes, trigger ketoacidosis in type 1 diabetes and accelerate the occurrence of known complications of diabetes. Although humoral and cellular response to vaccination might be hampered in persons living with diabetes, multiple studies have shown influenza vaccination to be effective and safe [30, 31].

**Fig. 1:**
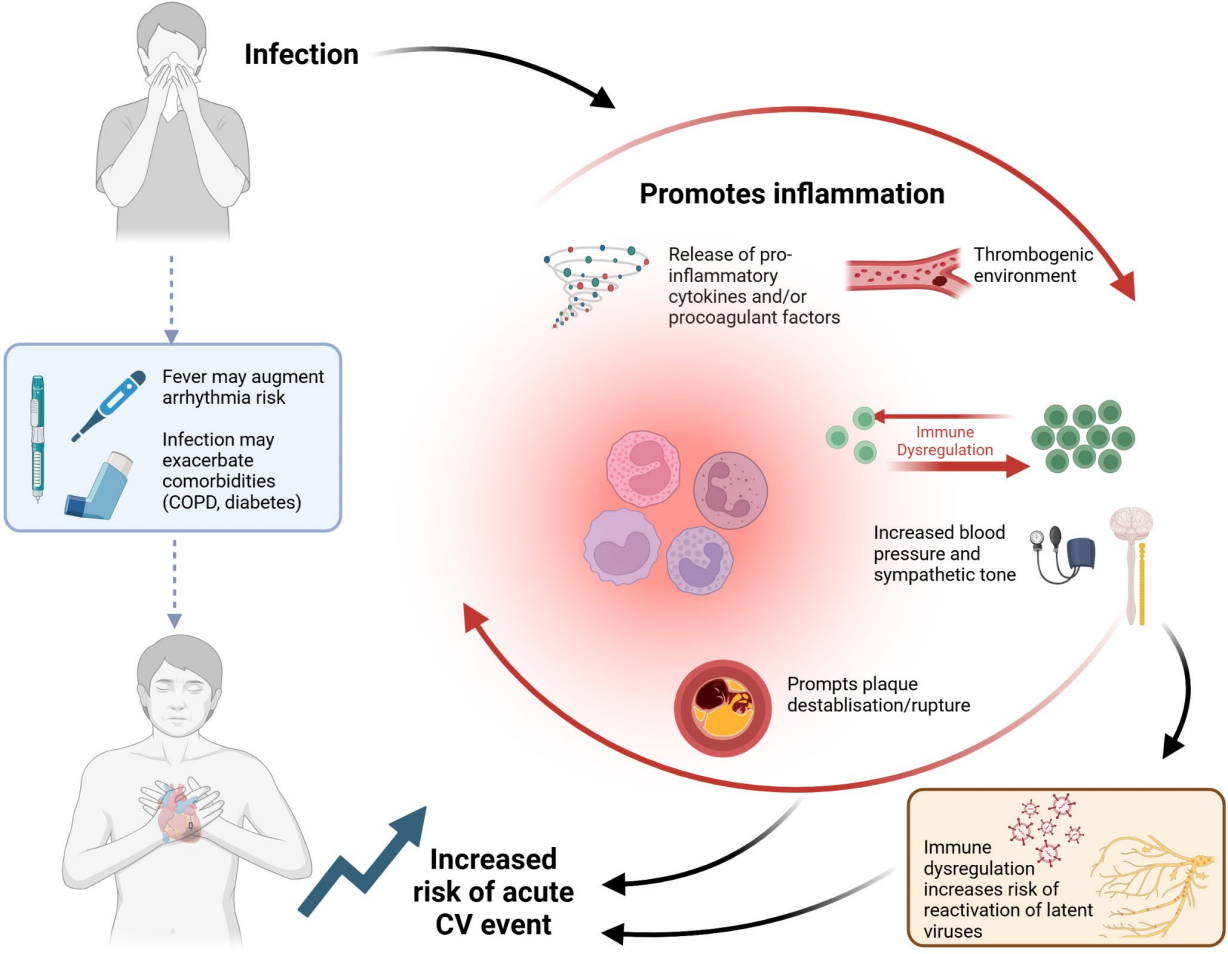
Mechanisms of increased cardiovascular risk during infection During acute infections, in particular respiratory infections (e.g. influenza, RSV, COVID-19, pneumonia), the immune system is activated, with release of proinflammatory cytokines and procoagulant factors, disrupting endothelial function and creating a thrombogenic environment and hypercoagulable state. This promotes systemic inflammation. In this context, immune dysregulation may promote increased sympathetic tone and high blood pressure. Taken together, these inflammatory conditions may promote plaque destabilization and rupture, causing an acute cardiovascular event. In addition, symptoms of infection, such as fever, may increase the risk of arrhythmia, while infection itself often exacerbates comorbidities such as chronic obstructive pulmonary disease, asthma or diabetes. The conditions of immune dysregulation may also increase the risk of reactivation of latent viruses Created in https://BioRender.com

The mechanisms linking infection (particularly respiratory infection) with increased risk of cardiovascular events are illustrated in Fig. 1.

Evidence exists to show that the complications of respiratory infections can be prevented by vaccination in patients with cardiovascular disease [32–34]. Based on this evidence, there are now consistent recommendations from all major professional societies for vaccination against the most common respiratory diseases caused by infections from bacteria such as *Streptococcus pneumoniae* and viruses that include influenza and SARS-CoV-2 [35–40]. Key vaccines recommended for older adults, and their cardiovascular benefits are summarized in the Table 1. Vaccines against RSV have only recently become available, and although recommendations are gradually being incorporated into national vaccine recommendations for older adults worldwide, very few recommendations exist from professional societies, as yet. Real-life studies reporting the impact of RSV vaccines on CV events are also lacking.

**Table 1:** Key vaccines recommended for older adults and their cardiovascular benefits

Vaccine	Target Population	Cardiovascular Benefits
Influenza	All adults ≥ 65 years old, adults with heart disease, diabetes, or other chronic conditions.	Reduces risk of influenza-related heart attacks, strokes, and cardiovascular complications
Pneumococcal vaccines (PCV20, PPSV23)	Adults ≥ 65 years old, adults with chronic heart disease or other risk factors	Prevents pneumonia and bloodstream infections, reducing inflammation that can trigger cardiovascular events
COVID-19	All adults, especially those ≥ 65 years old and with cardiovascular disease	Lowers risk of severe COVID-19, which is linked to both arterial and venous complications
Respiratory Syncytial Virus	Adults ≥ 60 years old, especially those with heart or lung disease	Reduces risk of severe RSV infections, which can worsen heart failure and COPD
Herpes Zoster	Adults ≥ 50 years old (age threshold for eligibility varies between countries)	May reduce risk of stroke associated with shingles and post-herpetic neuralgia

PCV20, 20-valent pneumococcal conjugate vaccine; PPSV23, Pneumococcal Polysaccharide Vaccine 23-valent; COVID, coronavirus disease; RSV, respiratory syncytial virus; COPD, chronic obstructive pulmonary disease

Herpes zoster is an infection caused by the reactivation of dormant varicella zoster virus (VZV). It is characterised by a painful, vesicular rash. Several pathological mechanisms have been suggested as possible explanations for the association between cardiovascular conditions and the risk of HZ, notably the role of immune system dysregulation and inflammatory pathways in various cardiovascular conditions [41–43]. Overall, immune system dysregulation not only plays a key role in the development of CV conditions, but also exacerbates the risk of HZ through mechanisms including immunosuppression and accelerated immunosenescence. The risk of HZ in patients with CV conditions has been widely studied and reported [44–49], with increases in risk ranging from a moderate 4% increase in patients with coronary artery disease [48], to a 25-fold increase in risk of HZ at one year after stroke [46]. Conversely, HZ is also known to increase the risk of certain CV events, such as stroke after HZ infection [50]. The risk of stroke was highest at 1 month, then decreased over time up to about a year [51], while the risk of myocardial infarction was highest within the first week [52]. The possible mechanisms explaining the increase in cerebrovascular and cardiovascular events after HZ infection include systemic inflammation, increased sympathetic tone and blood pressure, and altered immunological status, which, together, can promote vasculopathy [53–55]. A highly effective vaccine against HZ is available, namely a recombinant,non-live glycoprotein E subunit vaccine using the proprietary AS01_B_ adjuvant system [56, 57].

In summary, several common vaccine-preventable diseases exacerbate the risk of cardio- or cerebrovascular disease and conversely, are more likely to affect persons with cardio- or cerebrovascular disease. Preventing infectious diseases through vaccination is an easy and cost-effective means to prevent not only the burden of disease from the infections targeted, but also the possible repercussions on the cardiovascular system. It is crucial to improve the awareness of healthcare professionals and the general population, especially individuals living with cardiovascular conditions or risk factors, about the protective value of vaccines against cardiovascular and cerebrovascular events. In particular, addressing vaccine hesitancy among older adults is crucial for protecting this high-risk population from severe illness, hospitalization, and death due to vaccine-preventable diseases. Lack of information, misinformation, fear of side effects, and mistrust in healthcare providers can all contribute to hesitancy, making targeted education and outreach essential in this population. All healthcare professionals, including medical doctors, nurses and pharmacists, should proactively educate the community about the value of vaccines and contribute to improving vaccine uptake rates, namely by administering vaccines to all eligible individuals. Professional guidelines should provide more detailed evidence about the importance and benefits of vaccination and issue firm recommendations for immunization in patients with or at risk of cardiovascular disease.

## Data Availability

No datasets were generated or analysed during the current study.
